# DNA Damage-Induced RNAPII Degradation and Its Consequences in Gene Expression

**DOI:** 10.3390/genes13111951

**Published:** 2022-10-26

**Authors:** Juan Cristobal Muñoz, Inés Beckerman, Ramveer Choudhary, León Alberto Bouvier, Manuel J. Muñoz

**Affiliations:** 1 Instituto de Fisiología, Biología Molecular y Neurociencias (IFIBYNE-UBA-CONICET), Ciudad Universitaria, Buenos Aires C1428EHA, Argentina; 2 IFOM ETS—The AIRC Institute of Molecular Oncology, Via Adamello, 16, 20139 Milan, Italy; 3 Departamento de Fisiología, Biología Molecular y Celular, Facultad de Ciencias Exactas y Naturales, Universidad de Buenos Aires, Ciudad Universitaria, Buenos Aires C1428EHA, Argentina

**Keywords:** RNAPII degradation, DNA damage, gene expression, nucleotide excision repair, UV light

## Abstract

RPB1, the major and catalytic subunit of human RNA Polymerase II (RNAPII), is specifically degraded by the ubiquitin–proteasome system upon induction of DNA damage by different agents, such as ultraviolet (UV) light. The “last resort” model of RNAPII degradation states that a persistently stalled RNAPII is degraded at the site of the DNA lesion in order to facilitate access to Nucleotide Excision Repair (NER) factors, thereby promoting repair in template strands of active genes. Recent identification and mutation of the lysine residue involved in RPB1 ubiquitylation and degradation unveiled the relevance of RNAPII levels in the control of gene expression. Inhibition of RNAPII degradation after UV light exposure enhanced RNAPII loading onto chromatin, demonstrating that the mere concentration of RNAPII shapes the gene expression response. In this review, we discuss the role of RNAPII ubiquitylation in NER-dependent repair, recent advances in RPB1 degradation mechanisms and its consequences in gene expression under stress, both in normal and repair deficient cells.

## 1. Introduction

As the first step in gene expression, transcription is a tightly regulated process, and its correct progression is crucial for the normal development of every eukaryotic cell. RNA Polymerase II (RNAPII) is a multi-subunit complex that drives the expression of protein-coding RNAs and various non-coding RNAs. RPB1, the major and catalytic subunit of RNAPII, has been historically recognized as a unique factor due to the presence of repetitive heptads in its Carboxyl Terminal Domain (CTD). Extensive post-translational modifications of RNAPII’s CTD residues serve as a platform for several enzymatic complexes mainly involved in regulation of co-transcriptional pre-mRNA processing and chromatin remodeling [1,2]. In contrast to other DNA-dependent RNA polymerases, RPB1 is not only unique because of its particular CTD, but also because of its fate during the cellular response to DNA damage. Indeed, acute induction of DNA damage promotes the specific degradation of RPB1 in the proteasome [3].

Nucleotide Excision Repair (NER), the most versatile DNA repair system in human cells [4], is a multi-step process that deals with a wide variety of DNA lesions, including those induced by UV light exposure. DNA-damage recognition is initiated by two distinct sensing mechanisms, Global Genome repair (GG-NER) and Transcription-Coupled repair (TC-NER), both of which then converge in the usage of the same machinery to actually repair the lesion. In the GG-NER sub-pathway, damage recognition relies on Xeroderma Pigmentosum (XP) E and C (XPE and XPC) factors, which detect damage throughout the genome [5]. In contrast, TC-NER detects damage exclusively in the template strand of actively transcribed genes, where recognition is accomplished by an RNAPII complex unable to bypass the lesion [6,7]. To preserve the integrity of the genome, stalling of RNAPII favors the rapid recruitment of specific TC-NER factors that deal with the damage, favoring its repair. It was proposed quite some time ago that the fate of a stalled RNAPII either includes backtracking of the entire RNAPII complex, or alternatively, degradation of RPB1 [8]. In this sense, RNAPII degradation is thought to occur as a “last resort” in order to expose the lesion, only when backtracking is not possible [8]. More recently, it has been demonstrated that RNAPII stalling induces ubiquitylation of RPB1 K1268, with profound implications in lesion repair [9], RPB1 degradation and gene expression [9,10]. In this review, we discuss current knowledge on RNAPII degradation mechanisms and their global consequences on transcription, emerging determinants in the gene expression field.

## 2. Nucleotide Excision Repair

Multiple endogenous as well as exogenous sources of DNA damage continuously threaten DNA, causing an estimated number of 10^5^ DNA lesions per cell per day [11,12]. Since DNA is the only macromolecule that cannot be replaced, it must therefore be repaired. As an evolutionary response to DNA damage, cells have developed a collection of tightly regulated multi-step signaling pathways, the so-called DNA Damage Response (DDR) [13]. This orchestrated cellular response is in charge of sensing the DNA damage, signaling its presence and promoting its repair, and hence ensuring a highly coordinated response that preserves genome integrity. The DDR comprises several repair pathways including Nucleotide Excision Repair (NER), Base Excision Repair (BER), Mismatch Repair (MMR), Homologous Recombination (HR) and Non-Homologous End Joining (NHEJ), among others, all of which deal with different classes of lesions and have been extensively reviewed in [14].

NER is the most versatile repair mechanism cells have, given its ability to deal with a broad range of DNA damage types. This striking feature relies on its ability to recognize any helix-distorting DNA alterations, instead of having a specific set of effectors for every different type of DNA damage. Thus, NER deals with a plethora of lesions, including bulky DNA-adducts formed by chemical compounds such as 4-nitroquinoline, crosslinking agents such as cisplatinum, a particular type of oxidative damage—cyclopurines—and the damage induced by UV-radiation [4]. UVC irradiation mainly induces intrastrand crosslinking between two adjacent pyrimidines, and the two major DNA lesions formed are the Cyclobutane Pyrimidine Dimers (CPDs) and the 6-4 Pyrimidine-pyrimidone Photoproducts (6-4PPs) [15]. In the NER response, damage recognition is achieved by two different sub-pathways: Global Genome NER (GG-NER) and Transcription Coupled NER (TC-NER). GG-NER detects damage throughout the genome while TC-NER detects damage exclusively in the template strand of actively transcribed genes. Both detection systems converge in a single repair mechanism that involves the recruitment of the general transcription factor TFIIH, a transcription and repair complex containing factors needed for double helix opening (XPB/XPD), excision of the fragment containing the lesion (XPA, XPG and XPF), and gap-filling of the single stranded DNA (ssDNA) by replicative DNA polymerases [16].

## 3. Transcription-Coupled Recognition of the Damage

**Figure 1:**
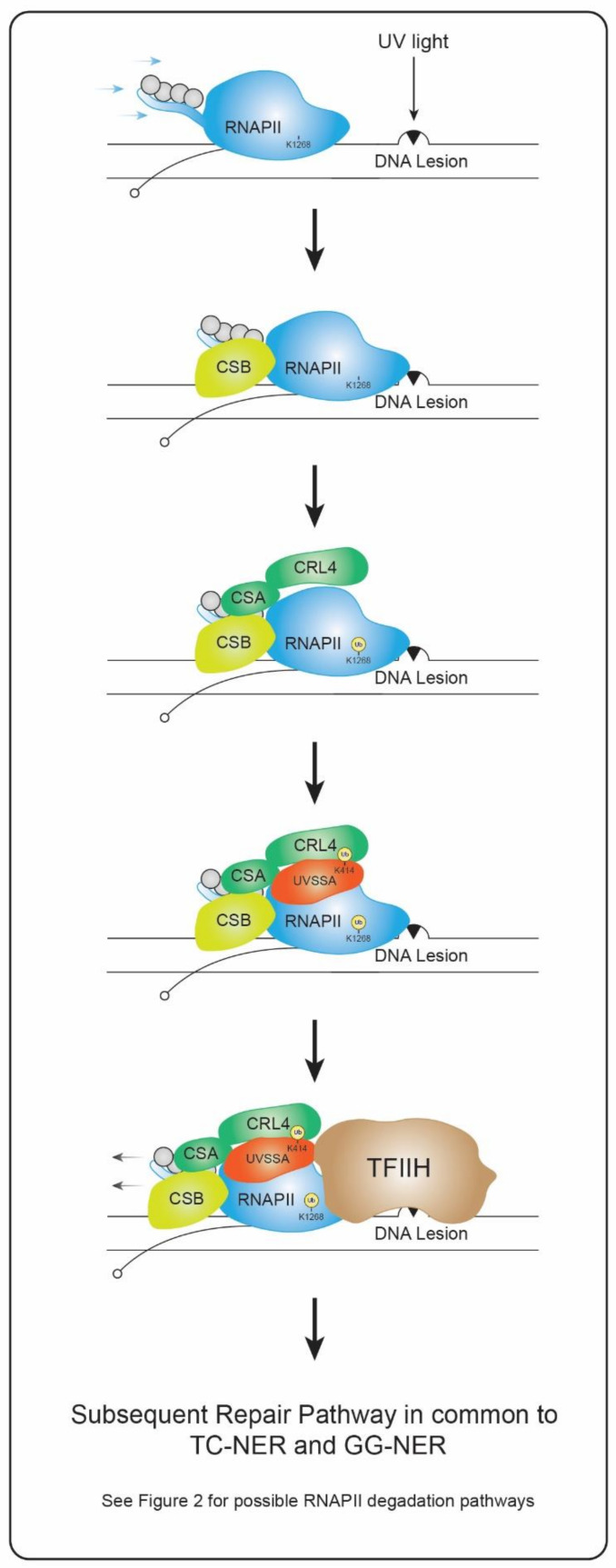
Model of Transcription-Coupled recognition of DNA damage. Stalling of RNAPII induces the recruitment of CSB and the E3 ligase complex CRL4CSA, which stands as the major contributor to RPB1 K1268 ubiquitylation upon UV, although other E3 ligases might be involved (see references [8,28,29]). Once RPB1 is ubiquitylated, UVSSA is recruited and ubiquitylated in its K414 residue, which facilitates TFIIH binding to the stalled complex. In order to expose the lesion and hand it over to TFIIH, the stalled RNAPII must backtrack, at least, a few nucleotides. Recruitment of TFIIH is the point at which GG-NER and TC-NER converge in the same mechanism in charge of completing the repair process. For more information regarding the fate of the ubiquitylated RPB1, see Figure 2.

An elongating RNAPII that stalls in front of a lesion functions as a damage recognition factor, initiating the preferential repair in template strands of actively transcribed genes through the TC-NER sub-pathway [6,7]. Both CPDs and 6-4PPs are recognized with the same efficiency through this sub-pathway, since both lesions represent an obstacle for an elongating RNAPII [17]. Stalling of RNAPII recruits the TC-NER specific factor Cockayne Syndrome B (CSB), which is an ATP-dependent 3′-5′ translocase that, under normal conditions, transiently interacts with the transcriptional machinery and, upon damage induction, gets persistently bound to the stalled RNAPII complex [10,18]. Studies on Rad26, the *Saccharomyces cerevisiae* orthologue of CSB, showed that after binding to the stalled RNAPII, and thanks to its DNA-translocase activity, Rad26 pushes RNAPII forward towards the damage in an attempt to bypass the lesion [19]. However, as already mentioned, UV-induced DNA damage cannot be bypassed by an elongating RNAPII, whose stalling stabilizes its interaction with CSB. As a consequence, CSB exposes a CSA-interacting motif (CIM) leading to the recruitment of Cockayne Syndrome A (CSA), part of the DDB1-CUL4-RBX1 (CRL4) E3 ubiquitin ligase complex [20] (Figure 1). In this sense, CSB-deficient cells are not only defective in CSA recruitment to the damage site [21], but also in TC-NER [9,10,17]. CSA, the substrate-recognition factor of the CRL4^CSA^ E3 ligase complex, binds to the stalled RNAPII-CSB complex, inducing its auto-ubiquitylation, ubiquitylation of CSB [22,23,24] and recruitment of the TC-NER specific factor UVSSA, which is mono-ubiquitylated in its K414 and is thought to interact with CSA through a CSA-interacting region (CIR) [20] (Figure 1). UVSSA in turn recruits USP7 [25], an enzyme with deubiquitylation activity that might stabilize CSB levels, although degradation of ubiquitylated CSB is not completely clear [22,25,26]. Nevertheless, UVSSA recruits TFIIH through a TFIIH-interacting region (TIR), which is a necessary step for efficient damage repair [20,27]. All together, these results suggest that CSB and CSA are recruited to a stalled RNAPII complex, promoting the recruitment of UVSSA, which is a factor needed for efficient TFIIH binding to DNA damage (Figure 1). In this regard, three-dimensional structures of TFIIH-XPC or TFIIH-UVSSA have been both identified, proving that recruitment of TFIIH is the point at which GG-NER and TC-NER converge in a common NER repair pathway [27].

Recruitment of TC-NER specific factors CSB, CSA and UVSSA must be a precise timely-regulated process that ensures the correct positioning of every factor in the repair complex needed for TFIIH binding and the subsequent repair of the DNA damage. However, TFIIH recruitment to damage sites does not solely rely on CSB, CSA and UVSSA, but also on RNAPII, as RPB1 K1268 ubiquitylation upon UV irradiation proved to be necessary for efficient TFIIH recruitment to the stalled complex [9]. In this sense, binding of UVSSA to the lesion site, which ultimately promotes its mono-ubiquitylation and TFIIH recruitment, was shown to depend on K1268 ubiquitylation [9]. In addition, ELOF1, a previously reported transcription-elongation factor needed for normal RNAPII elongation rates, has been recently involved in TC-NER [33]. ELOF1 is necessary for RPB1 ubiquitylation and efficient repair of damage, since ELOF1 KO mutant cells displayed an impairment on RNAPII K1268 ubiquitylation and UVSSA recruitment [21,34]. These results suggest that alongside CSB, CSA and UVSSA, RPB1 K1268 ubiquitylation and ELOF1 are relevant for proper damage recognition and TFIIH recruitment during TC-NER.

Upon TFIIH recruitment, and together with XPA, the actual presence of a lesion is verified in order to circumvent any futile repair reactions at damage-free sites [35,36]. This process involves the TFIIH DNA-helicase subunits XPB and XPD [37], which open and scan the region in search for any helicase-blocking lesions. Furthermore, it has been proposed that XPD’s helicase activity is specifically required for damage verification [38]. Once the presence of the lesion is confirmed, XPF and XPG endonucleases excise a fragment of 25–30 nucleotides along the strand containing the lesion, therefore exposing a ssDNA region on the undamaged strand, which is later used as a template by DNA polymerases and ligases to complete the repair process [16].

## 4. RNAPII Ubiquitylation

RPB1 ubiquitylation and degradation have been described more than 20 years ago [3,39], but the precise mechanism regulating these processes is not yet fully understood. However, K1268 has recently been identified to be the main residue involved in RPB1 ubiquitylation, since K1268R mutants displayed a prominent decrease in RPB1 ubiquitylation and degradation upon UV. However, a remaining mono-ubiquitylation is still observable in K1268R cells, hence suggesting that other residues can be ubiquitylated in response to damage [9,10]. In line with this, different E3 ligases have been implicated in RPB1 ubiquitylation [8,28,29], while nearly a complete suppression of RPB1 mono-ubiquitylation is observed when using a pan-inhibitor of Cullins, a family of E3 ligases [9,10]. In this sense, Cullin4-based CRL4^CSA^ is actually the major contributor to RPB1 K1268 ubiquitylation [9,20], as cells lacking CSA showed a drastic decrease in RPB1 ubiquitylation 3 h after UV [10]. Nevertheless, immediately after UV irradiation, CSA KO cells showed the same RPB1 ubiquitylation profile as wild-type cells, suggesting that many E3 ligases are involved [10]. In line with this, it has been proposed that NEDD4 first mono-ubiquitylates RPB1 and then Elongin_ABC_-Cullin5 poly-ubiquitylates it, targeting RPB1 for proteasomal degradation [40,41,42]. Moreover, BRCA1/BARD1 and Cull3-based ARMC5 were also proposed to act as RPB1 E3 ligases [43,44], suggesting that several steps of ubiquitylation/de-ubiquitylation may be necessary for a timely-controlled RPB1 eviction from chromatin and degradation [26]. The fact that many E3 ligases have been implicated in RPB1 ubiquitylation might be either an indicator of a multistep process controlling RPB1 ubiquitylation and/or multiple mechanisms controlling RNAPII fate, with different E3 ligase complexes involved in different scenarios. In this sense, it has been recently shown that UV irradiation induces degradation of promoter-bound RPB1 [30,31]. Considering that not all promoter-bound RPB1 molecules that are degraded have necessarily confronted a lesion, this process must be regulated in trans, in good agreement with the idea of multiple mechanisms involved in RPB1 ubiquitylation and degradation.

Moving beyond the E3 ligases involved, identification of RPB1 K1268 as the main residue ubiquitylated in response to UV has shed light on the regulation of the TC-NER mechanism and RPB1 degradation dynamics. K1268 ubiquitylation was shown to be necessary for efficient TC-NER, probably due to the fact that the binding of UVSSA and the subsequent recruitment of TFIIH was impaired in K1268R cells [9]. Supporting the role of RNAPII ubiquitylation in DNA repair, strand-specific ChIP-Seq experiments confirmed severe TC-NER deficiencies in K1268R cells, as well as in cells lacking CSB, CSA and UVSSA [9]. In this sense, prevention of RPB1 ubiquitylation resulted in longer chromatin-residency times of RNAPII and CSB, suggesting that eviction of RNAPII is relevant for efficient damage repair [10]. In this regard, although it is tempting to speculate that a stalled RNAPII may backtrack and resume transcription from the damage site after repair is completed, scarce evidence supports this idea. Dr. Aziz Sancar and colleagues [45] have shown that a stalled RNAPII complex is actually evicted from the lesion site, probably after dual incision by XPF and XPG. This model assumes that before eviction from chromatin, RNAPII first needs to backtrack and handover the lesion to TFIIH, in a process that has been proposed to depend on RPB1 K1268 ubiquitylation [9,46]. Moreover, several results showed that transcription restart occurs in a 5′-3′ wave from Transcription Start Site (TSS) after UV, and not from the lesion site [47,48], further strengthening the idea that RNAPII ubiquitylation and eviction from chromatin are needed for an orchestrated response upon DNA damage. Therefore, backtracking and eviction from chromatin may not be mutually exclusive mechanisms. Rather, the results reviewed in this section support a model in which a stalled RNAPII complex may first need to backtrack a few nucleotides, handover the lesion to TFIIH and only then be evicted from chromatin. Whether RNAPII eviction and degradation, or just eviction, are necessary to clear the lesion and favor its repair is not clear since, thus far, we just began to understand the role of RNAPII ubiquitylation in repair, chromatin removal and degradation.

## 5. RNAPII Degradation: Last Resort Model?

**Figure 2:**
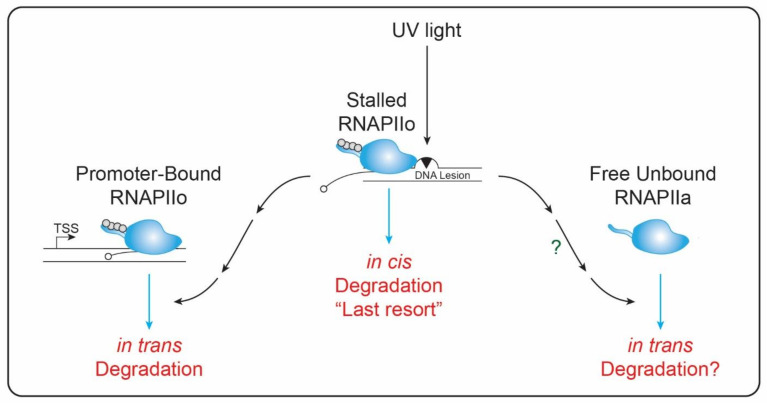
Possible RPB1 degradation pathways. RPB1 degradation upon DNA damage induction has been historically associated with a last resort, where RPB1 is degraded in cis to the DNA lesion, in order to facilitate access to TC-NER repair factors [8] (**center**). More recently, in trans degradation of promoter-proximal paused RPB1 has been proposed [30,31] (**left**) and VCP/p97 segregase independent degradation of RPB1 were also shown, opening the possibility of chromatin unbound RBP1 degradation [32] (**right**).

RNAPII stalling takes place only once the lesion reached the RPB1’s active site, where it remains occluded from the repair machinery [49,50]. Over the years, it was proposed that the fate of a persistently stalled RNAPII involves RPB1 degradation. This is known as the “last resort” model, in which RNAPII degradation occurs as a last resort in order to clear the lesion, hence allowing DNA repair [8] (Figure 2). This model supposes an in cis degradation, in which the stalled RNAPII is the one being targeted for degradation. Nevertheless, at least in theory, what is needed to clear the lesion is RNAPII eviction from chromatin, regardless of the fact that the actual evicted RPB1 molecule might be later degraded in the nuclear proteasome. RNAPII eviction likely depends on chromatin remodelers such as VCP/p97, an ATP-dependent segregase already reported to be necessary for chromatin eviction and degradation of elongating RNAPII molecules [51,52]. Alternatively, RNAPII degradation might occur at the damage site, when RNAPII is still attached to chromatin, as it has been recently proposed that the nuclear proteasome may directly interact with chromatin [53]. Whether this putative RPB1 degradation at chromatin represents a truly “last resort” remains to be determined. On the contrary, recent evidence favors the idea that RPB1 degradation may not necessarily be a last resort, as it has been shown that global degradation of promoter-bound RPB1 takes place upon UV-radiation, suggesting an in trans RPB1 degradation mechanism [30,31] (Figure 2). FRAP assays using GFP-RPB1 have shown degradation of promoter-bound RPB1 even in undamaged regions of the genome, with a reduction in P-Ser5 RPB1 levels [30], an RNAPII-CTD modification highly enriched in promoters [9,54]. Degradation of promoter-bound RPB1 proved to be dependent on the ubiquitin-dependent segregase VCP/p97. Therefore, promoter-bound RPB1 necessarily needs to be ubiquitylated before degradation, although the precise mechanism remains unknown. Strikingly, degradation of promoter-bound RNAPII showed no dependency on previously reported E3 ligases, further suggesting that multiple mechanisms may be involved in RPB1 proteasomal targeting [30]. Not surprisingly, an actively transcribing RNAPII was shown to be needed for promoter-bound degradation, since flavopiridol treatment, a CDK9 inhibitor that impedes RNAPII release from the promoter-proximal pausing [55], abolished RPB1 degradation [30]. Similarly, inhibition of GSK3, an RNAPII-CTD kinase involved in the regulation of transcription initiation and elongation [56], partially diminished RPB1 degradation [30]. These results suggest a model in which stalling of RNAPII in cis triggers degradation of promoter-bound RPB1 in trans. Moreover, it was recently shown that triptolide treatment induced RPB1 degradation in a VCP/p97-independent manner [32] which, together with the idea of an in trans degradation mechanism, opens the possibility that chromatin unbound RNAPII molecules could be degraded, even in the absence of VCP/p97 segregase activity (Figure 2).

In non-irradiated cells, acute depletion of SPT5 triggered a VCP/p97-dependent degradation of promoter-bound RPB1 [32]. SPT5 is part of the DSIF complex (SPT4/SPT5), which play key roles in transcription initiation, promoter-proximal pausing and elongation [57]. Phosphorylation of SPT5 by P-TEFb regulates the release of promoter-proximal paused RNAPII molecules while CDK9 knock-down prevented promoter-bound RPB1 degradation in SPT5-depleted cells [58]. It has been proposed that SPT5 can maintain the RNAPII complex in a closed elongating form, which is inaccessible to the repair machinery, thus repressing TC-NER [59]. Supporting this observation, it was shown that SPT5 competes with CSB for RNAPII binding [19,23], suggesting that RNAPII could switch from an elongating form when SPT5 is bound, to a TC-NER form when SPT5 is replaced by CSB in a stalled RNAPII complex. Therefore, it is possible to hypothesize that in SPT5-depleted cells CSB could be more persistently bound to the RNAPII complex, thereby inducing enhanced proteasomal degradation of RPB1. Hence, although the precise mechanism remains unknown, SPT5 could be involved in the regulation of promoter-bound RPB1 degradation, which might be specifically regulated in response to UV damage. Remarkably, RPB1 degradation in STP5 depleted cells was dependent on Cullin 3, but not on other previously reported E3 ligases, again strengthening the idea of multiple mechanisms controlling RPB1 ubiquitylation and degradation [32].

In summary, recently published studies suggest that chromatin-bound RNAPII could be degraded not only in cis but also in trans, provided that a stalled RNAPII molecule acts a lesion-sensor. Therefore, RPB1 degradation may not necessarily occur as a last resort, but rather as a fine-tuned mechanism that regulates the transcriptional response upon UV treatment (Figure 2).

## 6. Transcriptional Response to UV Treatment

Upon UV irradiation, cells undergo a complex transcriptional response involving an initial shutdown that lasts for several hours and is composed by multiple layers of regulation, followed by a transcriptional recovery once cells have dealt with the damage [60,61]. The transcriptional shutdown comprises an initial inhibition of elongation, with RNAPII being depleted from gene bodies within the first hour after irradiation and likely because of the physical block that DNA damage imposes on RNAPII-dependent transcription. However, it has been known for many years that stalling of RNAPII cannot solely account for the inhibition of transcription, as it has been shown that the elongation shutdown precedes a global inhibition of transcription initiation, which must necessarily be regulated in trans [47,61,62,63]. In this sense, cell-free extracts from damaged cells could efficiently inhibit transcription from undamaged DNA in vitro [61]. This is supported by recent observations showing that 45 min after UV transcription is restricted to the first 20 kb proximal to the promoter regions, while 3 h after, total depletion of RNAPII from chromatin is exhibited, even from the promoter regions [10].

However, a subset of short genes escape the initial elongation shutdown and is strongly and transiently upregulated in response to damage [64]. Considering that, immediately after UV irradiation, active RNAPII complexes run into DNA lesions with a chance that mainly depends on gene length and UV dose, short genes are less likely to be damaged and, therefore, more prone to escape the immediate transcription-elongation shutdown. As a consequence, the genes that escape the shutdown are particularly short, and many of them have been annotated as Immediate Early Genes (IEGs) and proto-oncogenes [10,64]. However, after the initial response, transcription of these short genes needs to be switched off, in order to avoid the unwanted effects of its persistent expression. Prevention of RPB1 degradation in K1268R mutant cells failed to turn off short genes compared to its wild-type counterpart, highlighting a direct role of RNAPII levels in dictating gene expression, where more RNAPII is simply associated with more expression [10]. Therefore, after UV irradiation, the initial elongation shutdown signals for the later global inhibition of initiation through a novel player: the reduction in RNAPII levels. Although the mechanism governing this process remains largely unknown, these results are consistent with the observed promoter-proximal degradation of RPB1 as a major hub of the transcriptional regulation in response to UV.

After the drastic transcriptional shutdown, cells recover their transcriptional profiles in a time-regulated process, and several mechanisms have been suggested to be involved in the regulation of the transcriptional recovery after UV. The transcription factor ATF3, encoded by one of the short genes upregulated rapidly after UV, binds to and downregulates the expression of thousands of genes, favoring the UV-induced transcriptional shutdown [62,65]. Importantly, it has also been demonstrated that ATF3 is specifically degraded at later time points, when transcription starts to recover [62]. Therefore, ATF3 has been proposed as a key regulator in the transcriptional response upon UV, as it peaks during the transcriptional shutdown but is degraded later during the recovery phase [62,66]. In this sense, it has been reported that knock-down of HIRA [67], a histone chaperone that targets ATF3 for proteasomal degradation, efficiently inhibits the recovery of RNA synthesis late after UV, highlighting the importance of degrading ATF3 in order to restore the transcriptional program. Nevertheless, K1268R mutant cells that fail to downregulate short genes still show high levels of ATF3, suggesting that different mechanisms simultaneously act to properly control gene expression under stress [10].

A remarkable feature of Cockayne Syndrome (CS) patient-derived cells is their inability to recover transcription after UV [60,68]. Therefore, CSA and CSB are essential not only for the transcription-coupled repair process itself, but also for the transcriptional recovery after UV exposure. In fact, ATF3 degradation depends not only on HIRA [66], but also on CSA and CSB, as it was shown that cells deficient in these factors fail to degrade ATF3 and, consequently, the RNA recovery phase following UV remains abolished [62,65]. In this regard, a recently identified pathway involving the PAF1 complex (PAF1C) and controlling the transcriptional response to UV also involves CSB and supports dual roles for this factor, first in transcription-coupled damage recognition and later in the transcriptional recovery [48]. During the recovery phase, CSB mediates the association of RNAPII and PAF1C, which promotes the release of promoter-proximal paused RNAPII and facilitates an efficient elongation, hence aiding in the recovery phase of transcription [48]. In a CSB dependent manner, PAF1C binds to the promoter region of thousands of genes that are transcriptionally downregulated by ATF3, promoting the transcriptional restart when ATF3 is degraded and cleared from promoters [48]. Therefore, CSB seems to be involved in the degradation of ATF3 and, at the same time, in the effective recruitment of PAF1C to promoters, therefore playing a crucial role in the recovery phase after UV. Overall, CSB is a major player in the transcriptional response to DNA damage since it is not only involved in the early phases after UV irradiation, as it favors DNA repair, but also later, where it promotes transcriptional restart.

As part of the transcriptional response to damage, UV treated cells also exhibit an immediate slowdown of RNAPII elongation rates, which proved to globally modulate the co-transcriptional processing of mRNAs [69]. The slowdown of RNAPII elongation rates persists for longer periods of time than the transcriptional shutdown, as it has been shown that transcription initiation recovers faster than elongation rates [47,63]. Not surprisingly, the slowdown of RNAPII elongation rates favors the usage of Alternative Last Exons (ALEs), thereby inducing the expression of different alternative-splicing isoforms [63]. A good example is ASCC3, a protein-coding gene whose alternative splicing is modulated in response to UV, shifting from a long protein-coding mRNA to a short ALE isoform, with non-coding functions. While it has been proposed that the protein encoded by the long isoform is involved in the maintenance of the transcriptional shutdown in response to UV irradiation, the short ALE isoform presents non-coding functions necessary for the later transcriptional recovery [63]. Consequently, the global slowdown of elongation rates is also physiologically relevant for the regulation of every step in the transcriptional response to UV irradiation, and therefore should be considered when studying the cellular response to UV treatment.

## 7. Conclusions and Future Perspectives

The cellular response to UV-induced DNA damage has been the subject of several studies for the last 50 years. However, recent identification of RPB1 K1268 as the main residue involved in RPB1 ubiquitylation and degradation brought a renewed interest in the gene expression field. It has been known for many years that immediately after UV, transcriptional elongation is impeded, as UV-induced DNA damage imposes a physical block to RNAPII elongation. After the initial elongation shutdown, a second step of transcriptional regulation takes place, as transcription initiation becomes globally inhibited. Impairment of RPB1 ubiquitylation and degradation resulted in more transcription, particularly in short genes, where the chance of being damaged is low. As a consequence, the drop in RNAPII levels emerges as a clear candidate to explain the initiation shutdown observed in UV treated cells.

Notably, it was recently demonstrated that RPB1 degradation takes place at promoter-proximal regions, further supporting the idea that RPB1 degradation is a tightly regulated process that mediates the second step of the transcriptional response to DNA damage. Likewise, it would not be surprising that multiple mechanisms are responsible for RPB1 degradation, depending on the RNAPII context and its interacting partners. Indeed, many E3 ligases have been considered capable of ubiquitylating RPB1 and different mechanisms have been shown to regulate its degradation, such as VCP/p97 dependent and independent pathways. The different mechanisms involved in RBP1 degradation most likely interact with each other to globally control the pool of RNAPII complexes, as signaling for in trans degradation likely depends on RNAPII lesion-stalling, where inhibition of RNAPII elongation decreased the observed degradation of promoter-proximal RNAPII molecules.

Regarding the recovery of RNA synthesis, gene expression analysis of *POLR2A*, the gene encoding for RPB1, as well as its pre-mRNA processing, mRNA translation and degradation, together with its epigenetic state, should also be considered when studying the transcriptional response to UV-induced DNA damage. Apart from the amount of RBP1, the recovery of RNA synthesis depends on ATF3 degradation and binding of PAF1C to promoters. Thus, while RPB1 degradation appears to be a major player in the initiation shutdown, once its levels start to recover, other mechanisms govern the transcriptional recovery. Therefore, recovery of RNA synthesis emerges as a key process in the transcriptional response to UV that might allow us to comprehend the complexity of CS patients. Indeed, CSA, CSB and UVSSA-deficient cells exhibit TC-NER deficiencies, but while UVSSA patients develop only mild clinical features such as increased photosensitivity [70], CSA and CSB patients display a more intricate array of features including not only photosensitivity but also growth failure and impaired development of the nervous system [71]. While CSA and CSB are both involved in TC-NER and in the transcriptional recovery after UV, UVSSA has only been related to TC-NER activity. Therefore, dissecting the multiple steps involved in the transcriptional response to UV is of outmost importance, not only to understand the precise mechanisms governing these processes, but also to explain and potentially modulate the transcriptional response in CS patients, for whom there is still no cure nor treatment.
